# The Effectiveness of Ablation Therapy for Atrial Fibrillation: A Systematic Review

**DOI:** 10.7759/cureus.43992

**Published:** 2023-08-23

**Authors:** Arturo P Jaramillo, Luisa Jaramillo, Rebeca R Briones Andriuoli, Jhoanny C Revilla, Javier Castells, Sabina Ibrahimli, Jossua L Villacres, Neyla Garzon Mora

**Affiliations:** 1 General Medicine, Universidad Estatal de Guayaquil, Machala, ECU; 2 Internal Medicine, Universidad Católica Santiago de Guayaquil, Guayaquil, ECU; 3 Pediatrics, Maimonides Medical Center, New York, USA; 4 Medicine, Universidad del Zulia, Maracaibo, VEN; 5 Medicine, Universidad Católica Santiago de Guayaquil, Guayaquil, ECU; 6 Cardiology, First Moscow State Medical University, Moscow, RUS

**Keywords:** cardiac arrythmia, cardiac ablation, atrial fibrillation (af), atrial fibrillation recurrence, non valvular atrial fibrillation

## Abstract

It is expected that the prevalence of atrial fibrillation (AF), the most prevalent cardiac arrhythmia among people aged 65 to 85, would be mostly classified using the CHAS2DS2-VASc approach for anticoagulation therapy. A high number of people in the entire world will be living with AF by 2030. Long-term follow-up data are sparse, although radiofrequency catheter ablation (CA) for symptomatic AF patients has the potential to be a curative therapy. Although women are referred later and less often than men, the outcomes following ablation are comparable across both genders. Health-related quality of life suffers from AF, and patients often find themselves less active as a result of their condition. AF may have a wide variety of symptoms and signs from the clinic's point of view. Women are more likely to exhibit symptoms than men; one reason for this is that women have an average QT interval that is 10-20 milliseconds longer than men, which is more likely to exacerbate tachycardia symptoms.

In search of medical databases for relevant medical literature, we looked at PubMed/Medline, the Cochrane Library, and Google Scholar. Ten publications were gathered after the papers were located, assessed, and qualifying criteria applied were used to select them. The finished articles were done to give an overview of the effectiveness of ablation therapy for AF. Some studies showed that there was no statistical significance between invasive and pharmacological treatments. Other research found no difference in the recurrence of atrial arrhythmia between pulmonary vein isolation (PVI) CA alone and PVI + enhancement magnetic resonance imaging (MRI)-guided fibrosis ablation in individuals with persistent AF. The oldest individuals in studies comparing CA to medical treatment for AF demonstrated no improvement in prognosis after CA. Also, complications from therapy and CA's efficacy in preventing future atrial arrhythmias were similarly low across all age groups. Based on the above, we concluded that more studies are required to establish the most effective approach to treating AF to apply it in daily practice and gain more knowledge about it.

## Introduction and background

Catheter ablation (CA) has witnessed a transformative shift in its therapeutic application for the management of AF. Before, CA was seen as a last resort for people who didn't respond to drug treatments and had severe symptoms. Now, CA is seen as a first-line treatment for people with different types of AF and different levels of severity [1,2]. Other improvements in understanding the important role of pulmonary vein isolation (PVI) in how well AF ablation works, as well as noticeable improvements in reducing procedural risks, have also helped this field move forward [2,3]. As advancements in equipment and expertise were made, the initial obstacles associated with the technique were successfully overcome [4]. This led to growing optimism among proponents, who envisioned the potential for providing enduring remedies to a subset of AF patients through CA. This optimism was particularly pronounced when CA was administered before the onset of irreversible atrial remodeling [4]. Left atrial fibrosis, a well-recognized characteristic of atrial myopathy, assumes a significant role in the underlying mechanisms of AF. With delayed enhancement magnetic resonance imaging (MRI), a strong link was found between the return of atrial arrhythmia after ablation and high levels of left atrial fibrosis at the start of the study [4,5]. This association remained statistically significant even after controlling for other potential confounding factors. In addition, it has been observed that the increased presence of residual fibrosis is strongly correlated with unfavorable outcomes following a medical procedure [4]. This finding emphasizes the significance of fibrotic myopathy in the perpetuation of an arrhythmogenic substrate. However, the efficacy of specifically targeting atrial fibrotic tissue during the ablation procedure to enhance the rates of recurrence of atrial arrhythmia in patients diagnosed with persistent AF has yet to be evaluated in extensive randomized clinical trials [2,4,5]. The challenge associated with the diagnosis of recurrent AF episodes, particularly in cases where they are asymptomatic, has led to an extensive period of deliberation regarding the actual duration of AF suppression after ablation [4,5].

Persistent AF ablation poses a significant challenge due to the high incidence of atrial arrhythmia recurrence following multiple treatment attempts [6,7]. There has been a close association of AF with coronary artery disease (CAD) and outcomes of CA are consistently worse in the subset of patients having underlying CAD [8]. In the realm of multicenter clinical trials, various approaches have been explored to enhance the efficacy of conventional PVI. These strategies encompass posterior wall ablation, supplementation with left-atrial roof line ablation, targeting of atrial rotors (areas characterized by re-entry phenomena), and targeting of complex fractionated atrial electrograms (sites exhibiting high-frequency electrical sources). However, despite these endeavors, none of these alternative techniques has thus far surpassed the effectiveness of conventional PVI [9,10]. Subsequent observational studies on CA have provided further insights into its efficacy in preventing AF recurrences, particularly among older individuals. These studies have demonstrated that CA offers relative advantages in terms of reducing AF recurrences in older age groups while maintaining relatively low complication rates [7,11]. The CABANA trial, also known as the catheter ablation versus antiarrhythmic drug therapy for atrial fibrillation trial, stands as a pioneering endeavor in the realm of medical research. It represents the first extensive prospective randomized trial that incorporates an extended follow-up period. The primary objective of this trial was to compare the efficacy of CA with that of drug therapy in a heterogeneous patient population encompassing various age groups and AF subtypes [11]. The CABANA study aimed to investigate the prolonged risk of AF recurrence in patients subjected to CA compared to those receiving pharmacological treatment. This investigation encompassed the assessment of both initial recurrence and the overall burden of AF. The initial results of the CABANA study revealed a noteworthy reduction of 48% in AF recurrence during a 48-month observation period [7,12].

## Review

Methodology

We did a systematic evaluation using free full-length papers and the Preferred Reporting Items Checklist for Systematic Review and Meta-Analysis (PRISMA) to describe our approach and results. 

Study duration

This review started on June 1st, 2023.

Search strategy

PubMed, Google Scholar, and Cochraine library were used to collect database using the following: (( "Atrial Fibrillation/classification"[Majr] OR "Atrial Fibrillation/complications"[Majr] OR "Atrial Fibrillation/drug therapy"[Majr] OR "Atrial Fibrillation/etiology"[Majr] OR "Atrial Fibrillation/genetics"[Majr] OR "Atrial Fibrillation/pathology"[Majr] OR "Atrial Fibrillation/prevention and control"[Majr] OR "Atrial Fibrillation/psychology"[Majr] OR "Atrial Fibrillation/rehabilitation"[Majr] OR "Atrial Fibrillation/surgery"[Majr] OR "Atrial Fibrillation/therapy"[Majr] )) AND ( "Atrial Fibrillation/classification"[Majr:NoExp] OR "Atrial Fibrillation/complications"[Majr:NoExp] OR "Atrial Fibrillation/drug therapy"[Majr:NoExp] OR "Atrial Fibrillation/etiology"[Majr:NoExp] OR "Atrial Fibrillation/genetics"[Majr:NoExp] OR "Atrial Fibrillation/pathology"[Majr:NoExp] OR "Atrial Fibrillation/prevention and control"[Majr:NoExp] OR "Atrial Fibrillation/psychology"[Majr:NoExp] OR "Atrial Fibrillation/rehabilitation"[Majr:NoExp] OR "Atrial Fibrillation/surgery"[Majr:NoExp] OR "Atrial Fibrillation/therapy"[Majr:NoExp] ) AND (( "Arrhythmias, Cardiac/classification"[Majr] OR "Arrhythmias, Cardiac/drug therapy"[Majr] OR "Arrhythmias, Cardiac/etiology"[Majr] OR "Arrhythmias, Cardiac/pathology"[Majr] OR "Arrhythmias, Cardiac/rehabilitation"[Majr] OR "Arrhythmias, Cardiac/therapy"[Majr] )) AND ( "Arrhythmias, Cardiac/classification"[Majr:NoExp] OR "Arrhythmias, Cardiac/drug therapy"[Majr:NoExp] OR "Arrhythmias, Cardiac/etiology"[Majr:NoExp] OR "Arrhythmias, Cardiac/pathology"[Majr:NoExp] OR "Arrhythmias, Cardiac/rehabilitation"[Majr:NoExp] OR "Arrhythmias, Cardiac/therapy"[Majr:NoExp] ) 

Eligibility criteria and study selection

To assess eligibility, two investigators carefully read the full title and content of each paper. We selected the latest literature and articles published in the past five years, including papers written in the English language, or if the full free-text English-language translation is available. Articles were excluded if the full text of the papers could not be retrieved. Articles focusing on AF and ablation therapy were strictly chosen. Gray literature and proposal papers were also not included.

Data management

Two independent writers evaluated papers based on titles and abstracts. Following that, significant abstracts were examined for a complete, free full-text examination. A third author evaluated the research after evaluating the chosen studies if there was any disagreement. Information from the relevant publications was then collected. The first author's name, type, year of publication, study design, and results were taken as priorities. Finally, duplicates were deleted.

Quality assessment

We used the assessment of multiple systematic reviews (AMSTAR) form and the Cochrane risk of bias assessment tools for clinical trials and for systematic reviews and meta-analyses. 

Results

**Figure 1:**
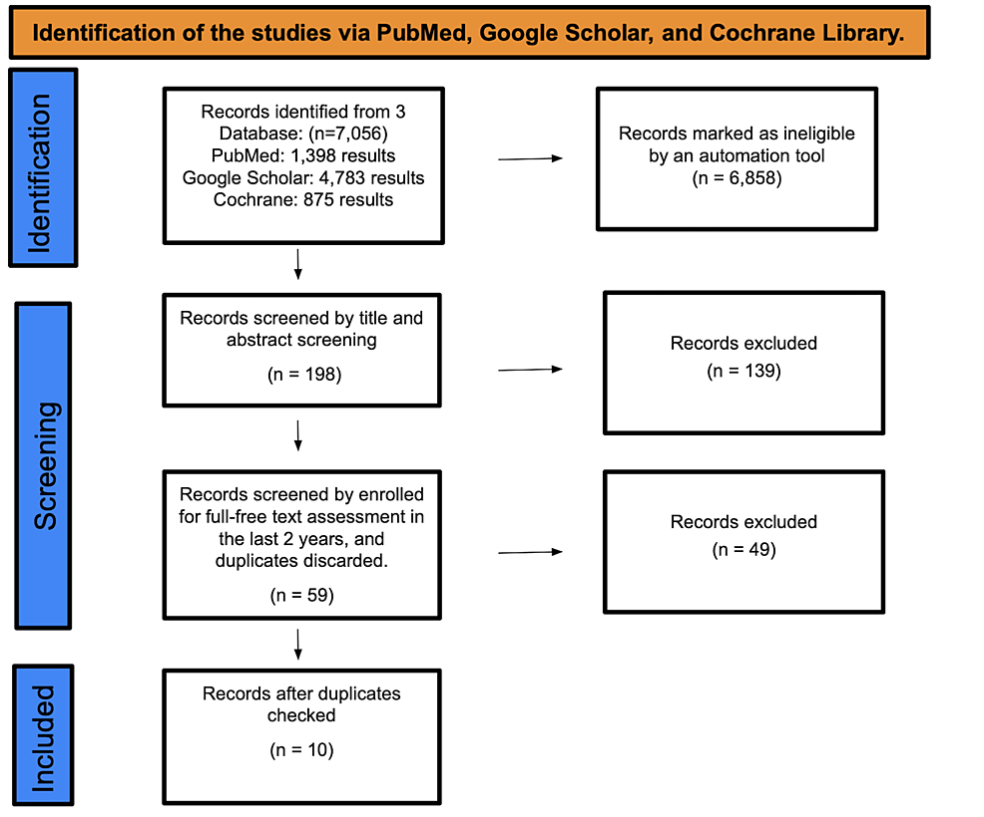
Search criteria and eligibility

A total of 7,056 studies were found after searching Pubmed, Google Scholar, and the Cochrane Library. A total of 6,858 were marked as ineligible by an automation tool. There were a total of 198 studies that underwent title and abstract screening, with 139 papers being discarded. The remaining 59 papers were chosen by full-free text evaluation in the previous two years, and after discarding duplicates, resulting in the elimination of 49 studies, only 10 studies were enlisted for the final collection of data (Figure 1).

See Table 1 for an in-depth description of the articles we decided to use.

**Table 1:** Table of data extraction RCT, randomized clinical trial; SRL, systematic review literature; AF, atrial fibrillation; PVI, pulmonary vein isolation; CABANA, catheter ablation versus antiarrhythmic drug therapy for atrial fibrillation; HR, hazard ratio; CA, catheter ablation; AFCA, atrial fibrillation catheter ablation; AADs, anti-arrhythmic drugs; ECG, electrocardiogram; PWI, posterior wall isolation; HFrEF, heart failure with reduced ejection fraction; AMSTAR, assessment of multiple systematic reviews

Author	Year of publication	Study design	Quality tool	Primary research	Outcome evaluation
Rashedi et al. [13]	2022	SRL and Meta-analysis	AMSTAR checklist	A comprehensive database search yielded 1057 results. 55 studies with complete texts were reviewed.	Observational studies show that discharge the same day following CA for AF is a safe and viable option. The risk of significant problems and readmission/emergency department visits is rather low and similar to that of patients hospitalized overnight.
Marrouche et al. [14]	2022	RCT	Cochrane risk of bias assessment tool	421 patients with one-arm treatment and 422 with two-arm treatment were randomly allocated to patients with persistent AF.	There was no difference between the groups in how often atrial arrhythmia came back with a [95% CI, 0.77-1.17; P=0.63] HR of 0.95.
Bahnson et al. [15]	2022	RCT	Cochrane risk of bias assessment tool	CA, or pharmacological therapy, was randomly assigned to AF patients 65 or older with one stroke risk factor.	It was found that CA outperformed pharmaceutical therapy in younger individuals.
Saglietto et al. [4]	2020	Meta-analysis	AMSTAR checklist	From their origin through April 30th, 2019, we examined the PubMed/MEDLINE and Embase databases. The first search, using the stated search parameters, yielded 1187 potential RCT studies.	The recent meta-analysis indicated that AFCA substantially reduces the risk of mortality, stroke, and heart failure hospitalization compared to AADs and rate control medicines.
Poole et al. [16]	2020	RCT	Cochrane risk of bias assessment tool	161 of 2204 enrolled patients (65.8% of whom did not get their randomized therapy) did not have post-90-day blanking data and were excluded from this analysis. This research used the CABANA trial's ECG recording monitor technology to track the remaining participants for recurrent AF.	CA reduced the recurrence of any AF by 48% and symptomatic AF by 51% over five years compared to medication. CA patients showed decreased AF loads regardless of their baseline AF type.
Thiyagarajah et al. [17]	2019	SRL and Meta-analysis	AMSTAR checklist	Three researchers compared PWI to PVI, whereas 16 and eight investigations reported acute procedural and clinical results, respectively.	In a significant number of patients, AF ablation may result in PWI and 12-month atrial arrhythmia freedom.
Richter et al. [18]	2019	RCT	Cochrane risk of bias assessment tool	Five smaller prospective RCTs and two newly published larger RCTs assessed the role and effectiveness of AF ablation in selected individuals with HFrEF.	When compared to medication treatment, CA offers a better approach to rhythm and symptom management, resulting in considerable clinical and functional benefits.
Packer et al. [19]	2019	RCT	Cochrane risk of bias assessment tool	The investigator-initiated, multicenter, randomized CA vs. ADD AF study has 126 sites in 10 countries.	When compared to conventional treatment, the CA method did not substantially lower the key composite outcome of mortality, debilitating stroke, major hemorrhage, or cardiac arrest in individuals with AF.
Daniel et al. [20]	2019	RCT	Cochrane risk of bias assessment tool	A double-blind, placebo-controlled trial of CA vs. medicine for the management of AF symptoms in 2204 patients younger than 65 with at least one risk factor for stroke	The average length of time that patients were followed up was 48.5 months. Of the 2204 patients who were randomly chosen, 1385 were men, 946 had paroxysmal AF, and 1256 had persistent AF. Of the 2204 patients, 1968 finished the study.
Wagner et al. [21]	2019	RCT	Cochrane risk of bias assessment tool	The CopenHeartRFA project randomized ablation patients 1:1 to extensive rehabilitation, which included a physical exercise regimen and psycho-educational counseling, or usual care.	In the study, there was a significant benefit in the treatment of patients with scores I-II.

Discussion

In this systematic review, we looked at all of the data collected for therapeutic atrial ablation for AF. We did this by putting together different research studies done in the field. Rashedi et al. found that planned and successful same-day discharge (SDD) had an acceptable success rate of 92.5%, low rates of major periprocedural problems (1.1% and 0.8%, respectively), and return rates of 4.9% and 5.0%, respectively. Also, there were no problems that were statistically different between the SDD and the overnight groups of patients. Notably, the number of major problems was a little bit lower in successful SDD than in planned SDD [13]. Sahashi et al. released a similar systematic review before, but they did not look at the success rate of SDD and did not try to use overnight stays as a control group. In other studies, Prasitlumkum et al. looked at this problem in a later meta-analysis. They tried to compare SDD and overnight groups in their study, even though this is a very likely way for selection bias to happen. This is because people with higher risks will be sent to the hospital, and people with lower risks will be picked for ambulatory SDD. This means that issues will be the same or even less in the SDD group [13]. Marrouche et al. found that MRI-guided fibrosis-targeted ablation with PVI helped people with chronic AF more than people with paroxysmal AF. PVs are well-defined parts of the body that can be targeted by many different types of ablation methods in a uniform, objective way. This is why different studies show the same success rates for PVI [14]. Fibrosis ablation, unlike PVI, is not regulated and does not have a set end goal for all operators. This means that aiming methods can be more flexible. In the group that had fibrosis-guided ablation and PVI, there was a higher chance of problems. This was mostly because there were more ischemic strokes. In the fibrosis-guided ablation + PVI group of this study, the rate of brain damage was similar to the rate of stroke seen in earlier published ablation studies that looked at other AF causes [14]. Harm to atrial tissue during ablation may make the left atrium work less well and may make it more likely that a blood clot will form at the ablation site, which raises the risk of an embolic stroke [14]. In Bahnson et al. RCT, older people are likely to face more short-term risks from the procedure, which may cancel out some of the long-term benefits of more effective AF control. However, in the CABANA study, none of the patients who were randomly given the ablation method died during the process, and major side effects that were not fatal were rare in both groups [15]. Also, AF patients who are older may have a more advanced, long-term disease with more atrial myopathy and changes. This idea means that it would be harder to treat AF in older people in a way that would keep the AF from coming back and that any benefits of treating AF would be smaller as a result [15]. Another reason why the effect of CABANA ablation treatment might change with age is that the rate of switching from drug therapy to ablation might change with age. If ablation lowers the chance of the main outcome, which is mostly death, then more crosses from the medicine arm should make the difference in the rates of the main outcome between the two arms smaller and make the treatment effect less strong [15]. Because of this, the lack of an age-related difference in death rates in the drug treatment arm and the resulting differences in the relative therapeutic benefits of ablation point to "the play of chance." Randomization only makes sure that the treatment groups are the same or that they can be moved, but it does not make sure that every possibly important trait is the same on both sides of a single study cohort [15].

Saglietto et al. found that atrial fibrillation catheter ablation (AFCA) was better than medical treatment at lowering hard clinical measures, with a 38% decrease in death, a 37% decrease in stroke, and a 36% decrease in stay for heart failure over a median of 3.5 years. AFCA is now a rhythm control option for people with AF who still have symptoms after getting the right medicine to control their heart rate [4]. In comparison, the improved efficiency and safety of AFCA in keeping sinus rhythm compared to anti-arrhythmic drugs (AADs) raises worries about the possible predictive effects of current rhythm management methods, either with AFCA alone or with a combination approach (AFCA + AADs). The catheter ablation for atrial fibrillation with heart failure (CASTLE-AF) trial showed that AFCA decreased the main goal of all-cause death or hospitalization in heart failure patients compared to drug treatment [4]. Based on published RCTs and meta-analyses comparing AFCA to conventional care in people without heart failure, there was no change in life. It should be noted, though, that only four studies were included in the second analysis. These studies were not strong enough to find differences in survival because they had a small number of patients (ranging from nine to 24 months, with a median of 12 months). Also, the follow-up time was short (from nine to 24 months, with a median of 12 months), and most importantly, the number of events that were looked into was very low (six and seven, in the pooled ablation and medical therapy cohorts, respectively) [4]. Poole et al. found that CA was linked to a 50% drop in the first return of AF, atrial flutter (AFL), or atrial tachycardia, no matter if the first recurrence caused symptoms or not. The second important finding was that having AFL or AT as the first recurrent arrhythmia after treatment had little effect on first recurrent atrial tachyarrhythmias [16]. The third important result was that the AF load, which was measured by the average percentage of time people were in AF during the Holter monitor, was much lower in patients who were treated with ablation than in patients who were treated with medicine for the whole five-year follow-up period. No matter what kind of AF a person had at the start (paroxysmal, persistent, or persistent/long-standing chronic), the effects were the same. Patients who had persistent or extended persistent AF at the start had a bigger drop in the amount of AF [16]. Poole et al. said in another study that CABANA patients with either untreated or undertreated AF, whether the AF was paroxysmal, continuous, or had been going on for a long time, could be included. Together, these factors, the longer follow-up, and the higher number of patients selected made it possible to test AFCA's effectiveness in preventing AF from coming back in a large group of AF patients [16]. Thiyagarajah et al. described another treatment as an immediate medical end goal for AF ablation. This treatment can be reached by a large number of patients and is not likely to cause big problems linked to the operation. The pooled values of 65.3% overall and 61.9% in the subgroup with persistent AF for 12 months without AF were close to those found in previous meta-analyses of pulmonary vein separation [17]. RCTs that looked at how posterior wall isolation (PWI) vs. PVI affected the return of arrhythmia came up with confusing results that could not be put together in a way that made sense [17]. Also, most of the patients in two of the three RCTs had paroxysmal AF, while those with persistent or long-term persistent AF may see the benefits of PWI more clearly [17].

## Conclusions

Based on the data from RCTs, meta-analyses, and systematic reviews, there is currently no accurate statistically significant evidence to determine the most effective and safe therapeutic approach for AF. This includes comparing AFCA with AADs, fibrosis ablation with PVI versus PVI CA alone, and other similar approaches. Therefore, it is recommended to conduct additional RCTs that specifically compare different treatments, including both pharmacologic and invasive approaches, to determine the most effective therapy for treating AF.
